# 329 Global Reach and Engagement of the SHEA International Ambassadors Program: Results of a Cross-Sectional Participant Survey

**DOI:** 10.1017/ash.2026.10674

**Published:** 2026-06-23

**Authors:** Hillary Spencer, Leah Vanham, Shana Altman, Julie Dow, Mike Bierman, Christopher Vogt, Andrea Statsholt, Kristin Campbell

**Affiliations:** 1 Illinois Department of Public Health; 2 Jackson County Health Department; 3 IDPH; 4 IL Department of Public Health

## Abstract

**Background:** Contaminated endoscopes are a recognized source of nosocomial transmission of infection. Endoscope-associated Salmonella transmission was once common, however, as a result of stringent reprocessing guidelines, including high-level disinfection, a 2018 review indicated that no cases were reported since 1987. **Methods:** In July 2024, the Illinois Department of Public Health identified and notified the local health department (LHD) of a cluster of three Salmonella Enteritidis (SE) cases from the same jurisdiction that were related by whole genome sequencing (WGS). The LHD investigated the cluster, as well as other Salmonella cases that were pending WGS, to identify a common source. The investigation included case interviews, interviews of patients with a common exposure, medical record reviews, isolate serotyping and whole genome sequencing (WGS), scope culture, and site visits for Infection Control Assessment and Recommendations (ICAR). **Results:** Index case reported symptoms including fever, nausea, vomiting, and urgent diarrhea. Two weeks prior to symptom onset, the case-patient had an esophagogastroduodenoscopy (EGD) and colonoscopy at an outpatient gastroenterology and endoscopy center. 56 patients had EGDs performed with the same scope, 48 of whom were interviewed regarding gastrointestinal symptoms post procedure. Six patients reported experiencing symptoms following their procedure, five of whom were already included in the investigation. The sixth case tested positive for Salmonella Newport and was excluded. Seven total cases of Salmonella were identified as associated with this investigation with illness onset between May 3, 2024, and September 20, 2024. Six were confirmed to be SE and closely related (0 SNPs) by WGS; one specimen was not sent to the state lab for WGS. ICAR identified several opportunities for improvement however there was not a clear gap in the disinfection or other processes that were observed. Namely, the scope passed the facility’s leak test. Scope culture was negative for Salmonella and other gram-negative organisms. The scope was sent to a third-party endoscope repair company; it did not pass their leak test, and the scope was refurbished. The facility planned to prospectively have all scopes inspected routinely. Staff was also retrained by the consultant company on reprocessing procedures. **Conclusion:** Scope-associated Salmonella transmission may be rare, but it is not entirely bygone. Cases may be under-reported if symptoms are attributed to the procedure itself or the underlying condition which prompted the procedure or are self-resolving and do not lead to diagnosis. Because scopes are complex in design and require fidelity to strict reprocessing, prospective maintenance routines, and re-training and auditing of reprocessing processes may be prudent.

